# Prolactin Modulates the Proliferation and Secretion of Goat Mammary Epithelial Cells via Regulating Sodium-Coupled Neutral Amino Acid Transporter 1 and 2

**DOI:** 10.3390/cells13171461

**Published:** 2024-08-30

**Authors:** Xiaoyue Ma, Hanling Liu, Wentao Li, Jianguo Chen, Zhenliang Cui, Zixia Wang, Changmin Hu, Yi Ding, Hongmei Zhu

**Affiliations:** 1 College of Veterinary Medicine, Huazhong Agricultural University, Wuhan 430070, China; xyma1998@163.com (X.M.); lhl414@webmail.hzau.edu.cn (H.L.); wentao@mail.hzau.edu.cn (W.L.); chenjg@mail.hzau.edu.cn (J.C.); 17740621016@163.com (Z.W.); hcm@mail.hzau.edu.cn (C.H.); dingyi@mail.hzau.edu.cn (Y.D.); 2 Ningbo Sansheng Biological Technology Co., Ltd., Ningbo 315000, China; zlccl2004@163.com

**Keywords:** PRL, SNAT1/2, goat mammary epithelial cells, STAT5 signaling, proliferation, lactation

## Abstract

The prolactin (PRL) hormone is a major regulator of mammary gland development and lactation. However, it remains unclear whether and how PRL contributes to mammary epithelial cell proliferation and secretion. The Boer and Macheng black crossbred goats are superior in reproduction, meat, and milk, and are popular in Hubei province. To elucidate the mechanisms of PRL on mammary growth and lactation, to improve the local goat economic trade, we have performed studies on these crossbred goats during pregnancy and early lactation, and in goat mammary epithelial cells (GMECs). Here, we first found that the amino acid transporters of SNAT1 and SNAT2 expression in vivo and in vitro were closely associated with PRL levels, the proliferation and secretion of GMECs; knockdown and over-expression of SNAT1/2 demonstrated that PRL modulated the proliferation and lactation of GMECs through regulating SNAT1/2 expression. Transcriptome sequencing and qPCR assays demonstrated the effect of PRL on the transcriptional regulation of SNAT1 and SNAT2 in GMECs. Dual-luciferase reporter gene assays further verified that the binding of the potential PRL response element in the SNAT1/2 promoter regions activated SNAT1/2 transcription after PRL stimulation. Additionally, silencing of either PRLR or STAT5 nearly abolished PRL-stimulated SNAT1/2 promoter activity, suggesting PRLR–STAT5 signaling is involved in the regulation of PRL on the transcriptional activation of SNAT1/2. These results illustrated that PRL modulates the proliferation and secretion of GMECs via PRLR–STAT5-mediated regulation of the SNAT1/2 pathway. This study provides new insights into how PRL affects ruminant mammary development and lactation through regulation of amino acid transporters.

## 1. Introduction

Prolactin (PRL) plays a major role in the development and secretion of ruminant mammary glands. In the embryonic stage of mammary gland development, PRL increases the activity of fetal mammary stem cells and produces the initial ductal structure of the gland at birth [1]. During late puberty, PRL, progesterone, and estrogen co-regulate the lobular buds branching out from the ductal system [2]. Upon pregnancy, PRL combines with progesterone, placental prolactin, and local growth factors to produce alveoli and accelerate the proliferation of lobular alveolar epithelial cells. Furthermore, postpartum PRL, in the context of decreased progesterone, stimulates the milk protein gene expression as well as maintains milk secretion. To modulate the physiological processes, PRL can regulate the expression of downstream genes through the Janus kinase-signal transducer and activator of transcription 5 (JAK-STAT5) signaling pathway. For instance, PRL regulated the expression of L-type amino acid transporter 1 (LAT1) through the STAT5 signaling pathway to affect the milk proteins in cows [3], and PRL played a role in premenopausal breast carcinogenesis through the JAK-STAT pathway [4]. Goats are an important source of milk and are found worldwide because of their adaptability to the environment and lower production costs compared with cows. According to Food and Agriculture Organization of the United Nations (FAO) data for 2024, the world’s goat population is estimated to be 1.14 billion, of which about 90 percent come from Asia and Africa. In China, the goat population is estimated to be about 130 million, making it one of the largest producers of goat milk and meat [5,6]. Boer goats are a world-famous meat-type goat breed that emerged from Africa and have been introduced into many countries to improve local breeds. In Hubei province in China, Macheng black goats are a popular local goat breed that offers meat and milk to people. The crossbreeding between Boer goats and Macheng black goats is prompted in Hubei province to improve the reproductive performance or economic traits of local breeds. This crossbred goat breed has its advantages in adaption to local climatic conditions, in its high milk yield, and good meat quality, making up a large part of the local goat population and being economically important for local agriculture [7,8,9]. In addition, ewes mating at eight months old exhibited a higher reproductive rate than those of adult ewes when stimulated by males [10]. These factors make the Boer and Macheng black crossbred goats representative models for studying the mechanisms of PRL regulating mammary lactation and clarifying the milk traits of Boer and Macheng black crossbred goats in Hubei province.

During the periods of pregnancy and lactation, the ruminants need a high demand for amino acids in the mammary gland to prepare for cell proliferation and secretion. Nevertheless, the uptake of amino acids by the cell is determined by amino acid transporters which can transport amino acids from the extracellular compartment into the cell [11]. Studies have demonstrated that the amino acid transporter system A, including sodium-coupled neutral amino acid transporter (SNAT) 1, 2, and 4, co-transported a wide range of small neutral amino acids, such as glutamine and alanine, into the mammary gland [12,13,14]. SNAT1 and SNAT2, encoded by *SLC38A1* and *SLC38A2*, are characterized as the classical system A transporter which are ubiquitous in mammalian cells and play vital roles in many tissues such as the mammary gland, brain, and placenta [15,16,17]. SNAT1 is a key contributor to system A activity in the second trimester of pregnancy and in human cytotrophoblast cells [18]; SNAT2 provides efflux substrates for other amino acid transporters such as amino acid hetero-exchanger system L, facilitating the uptake of branched-chain amino acids which activates the target of rapamycin (TOR) protein kinase pathway to accelerate protein synthesis and cell proliferation. Up-regulation of SNAT1/2 expression is mediated by different pathways. In the mammary gland, liver, brain, or placenta, SNAT1/2 are activated by glucagon via the cAMP/PKA pathway which then phosphorylates the transduction factor of the cAMP response element-binding protein (CREB), allowing the CREB to bind to the cAMP response elements (CRE) in the SNAT1/2 promoter regions [19]. Placenta-specific SNAT2 knockdown in mice reduced recent fetal and placental weights, fetal viability, and trophoblast plasma membrane SNAT2 protein abundance [16]. In undifferentiated neural progenitor cells, theanine accelerated cell proliferation and subsequent neuronal differentiation through a mechanism associated with up-regulation of the SNAT1 gene [20]. In another study, SNAT1 regulated the development of hepatocellular carcinoma by modulating phosphatidylinositol 3-kinase (PI3K)/protein kinase B (AKT)/mammalian target of rapamycin (mTOR) signaling via glutamine-mediated energy metabolism [21].

Researchers found that the SNAT1/2 mRNA levels in the rat or mouse mammary gland were progressively increasing during pregnancy and early lactation [22,23]. A further study in rat mammary gland explants found a positive correlation between SNAT2 expression and PRL and that PRL provided amino acids to glands via SNAT2 to synthesize milk proteins to support lactation [24]. Similarly, in mammary carcinoma cell lines which expressed PRLR, researchers found that PRL stimulated the expression of SNAT1/2. In cows, knockdown of PRLR or STAT5 with siRNAs significantly reduced PRL-stimulated LAT1 expression and milk production [3]. PRLR knockout in female mice mammary glands exhibited a reduction in the uptake of the amino acids transported by SNAT1/2 and an impairment of mammary development or lactation [25]. Remember that the endocrine regulation of mammary gland development and lactation in ruminants is crucial for milk production and the economies of many countries. We initially hypothesized that PRL modulated the proliferation and lactation of goat mammary epithelial cells (GMECs) through the PRLR-STAT5 pathway, while regulating downstream genes of SNAT1/2. Utilizing the RNA interference or overexpression technique, we directly verified the importance of SNAT1/2 in PRL promoting GMECs’ proliferation and secretion. Through RNA-sequencing and dual-luciferase reporter gene assays, we identified the effect of PRL on the transcriptional activation of SNAT1/2, and we explored whether the PRLR-STAT5 pathway mediated this process. Overall, our findings yield new insights into how PRL modulates ruminant lactation by regulating the mammary expression of amino acid transporters.

## 2. Materials and Methods

### 2.1. Animals and Sampling

**Figure 1:**
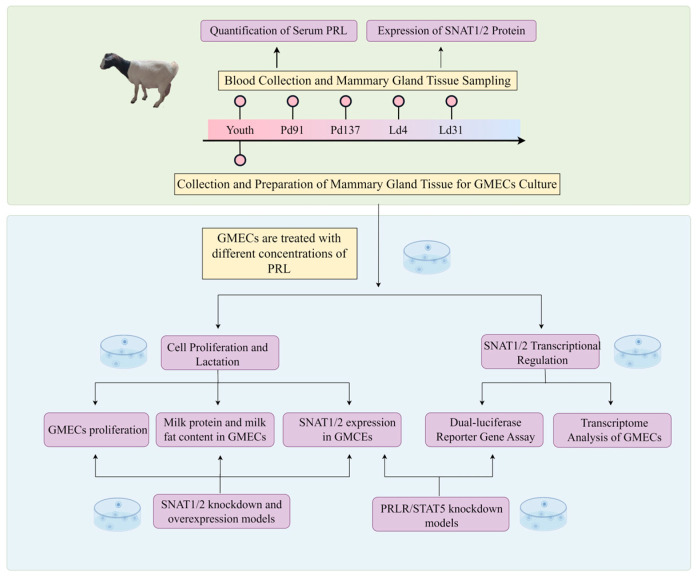
The schematic diagram of the whole experiment. Youth: The goats are in the stage of puberty; Pd91: The goats are in the stage of pregnancy, day 91; Pd137: The goats are in the stage of pregnancy, day 137; Ld4: The goats are in the stage of lactation, day 4; Ld31: The goats are in the stage of lactation, day 31; PRL: prolactin; SNAT1/2: sodium-coupled neutral amino acid transporter 1/2; GMECs: goat mammary epithelial cells (diagram by Figdraw).

All goat experiments were performed in accordance with the guidelines and regulations of the Huazhong Agricultural University and were approved by the Animal Care and Use Committee of Huazhong Agricultural University (ID number: HZAUGO-2020-001). We obtained the eight-month-old female Boer and Macheng black crossbred goats from the Hubei Academy of Agricultural Sciences, Wuhan, Hubei Province, China. The body weight of each goat was controlled at 20–30 kg. The whole experiment was performed in Wuhan, Hubei Province, which is located at the north latitude 29°58′–31°22′, east longitude 113°41′–115°05′, and with an altitude of 50 m above sea level. During this study, the animals were housed and fed in a controlled environment with a temperature of 22 °C, 50% relative humidity, a 12-h photoperiod, and with free access to water. Then, these goats were mated for their first pregnancy. The tissues were obtained from the mammary gland of the goats at the stage of puberty, pregnancy day 91 (Pd91), Pd137, lactation day 4 (Ld4), and Ld31 after being anaesthetized by 0.01 mL/kg Sumianxin (Veterinary Research Institute, Jilin, China) in a sterile operating room as previously reported [26]. The mammary tissue was processed for cell culture, mRNA, or protein extraction (Figure 1).

### 2.2. Cell Isolation and Culture

We isolated GMECs according to the protocol of a previous study [26]. Briefly, the tissue was collected from the mammary gland of the 8-month-old goats. Firstly, the tissue was washed with water and phosphate buffered saline (PBS), respectively, until the liquid was clear. Then, the tissue was immersed in 75% absolute ethanol for 3 min to disinfect it. Thirdly, the tissue was gradually cut into 1–3 mm^3^ pieces and washed with PBS several times to remove the impurities and tissue secretions. Finally, the clean tissue blocks were attached to 60-mm culture dishes with a distance of 1 cm and cultured in an incubator at 37 °C and 5% CO_2_ for 4 h. After the incubation, the adherent tissue sections were cultured in DMEM/F12 medium containing 12% fetal bovine serum (Hyclone, Logan, UT, USA). About 3–5 days later, the tissue culture medium was replaced with fresh culture medium. About 10 days after the culture, mainly two cell types grew out from the tissue sections: mammary epithelial cells and fibroblasts. We purified GMECs from fibroblasts by different time of TE (0.25% trypsin/0.05% EDTA) digestion. All experiments were conducted using GMECs within 6 passages.

### 2.3. Hormone Administration

To evaluate the effect of PRL on the GMECs’ proliferation and secretion, the cells were stimulated with multiple concentrations of PRL (ab269220, Abcam, Cambridge, UK). For the cell proliferation assay, the cells were divided into eight groups according to the concentrations of PRL: 0 ng/mL PRL group, 20 ng/mL PRL group, 50 ng/mL PRL group, 100 ng/mL PRL group, 200 ng/mL PRL group, 400 ng/mL PRL group, 800 ng/mL PRL group, and 1600 ng/mL PRL group. For the cell secretion assay, the cells were inducted for secretion by the induction system containing PRL, 10 ng/mL epidermal growth factor (EGF) (SRP3027, Sigma, Buchs, Switzerland), 1× insulin-transferrin-selenium (ITS) (I3146, Sigma, Switzerland), and 1 μg/mL hydrocortisone (HC) (R011855, Rhawn, Shanghai, China). Cells were divided into six groups according to the concentrations of PRL: control group, induced lactation system (IL) (EGF + ITS + HC) group, IL + 2500 ng/mL PRL group, IL + 5000 ng/mL PRL group, IL + 7500 ng/mL PRL group, and IL + 10,000 ng/mL PRL group. Cells were administered hormones for 12 h, 24 h, or 48 h for cell proliferation tests or were harvested for mRNA and protein.

### 2.4. Cell Proliferation Analysis

Cell viability was analyzed by Cell Counting Kit-8 (CCK-8, Biosharp, Wuhan, China). Cells were seeded into 96-well microplates. Then, the cells were treated with 0 ng/mL, 100 ng/mL, 200 ng/mL, 400 ng/mL, and 800 ng/mL PRL, respectively. After 48 h, the CCK-8 reagent was added to each well for 2 h. The absorbance at 450 nm was analyzed using a microplate reader (BMG LABTECH, Offenburg, Baden-Wurttemberg, Germany). The proliferation of cells was dependent on the absorbance.

5-Ethynyl-20-deoxyuridine (EdU) staining was used to analyze the GMECs’ proliferation according to BeyoClick^TM^ EdU Cell Proliferation Kit (C0075S, Beyotime, Shanghai, China). Briefly, GMECs were treated with different concentrations of PRL (0, 100, 200, 400, and 800 ng/mL) for 48 h. Then, the cells were incubated with EdU (20 mmol/L) and were fixed with 4% paraformaldehyde. The staining was finally visualized with a microscope.

### 2.5. Quantitative Real-Time PCR

Briefly, total RNA was extracted from each sample using AG RNAex Pro Reagent (Accurate Biology, Changsha China). RNA concentrations were measured using a NanoDrop 2000c spectrophotometer (NanoDrop Technologies, Wilmington, DE, USA). Reverse transcription was performed with a PrimeScript Reagent Kit with gDNA Eraser (Accurate Biology, Changsha, China). The qPCR was performed using SYBR Green Pro Taq HS Premix (Accurate Biology, Changsha, China) in a Roche LightCycler 96 real-time PCR system (Roche Diagnostics GmbH, Mannheim, Germany). Primers used for amplifying *SNAT1*, *SNAT2*, *BLG*, *CSN*, *PRLR*, *STAT5*, and *β*-actin are shown in Table S1. Expression of all genes was normalized using the geometric mean of endogenous genes (*β*-actin). The relative RNA expression levels were calculated by the 2^−ΔΔCT^ method, where ΔΔCt = ΔCt_1_ (experiment group) − ΔCt_2_ (control group), and ΔCt = Ct_targetgene_ − Ct_β-actin_.

### 2.6. Western Blotting

The cells were treated with RIPA lysis buffer (Beyotime, Shanghai, China) which contained 0.5 mM phenylmethylsulfonyl fluoride (Beyotime) to collect proteins. Protein concentrations were determined by the bicinchoninic acid method (Biosharp, Wuhan, China). Then, the protein samples were separated and were transferred to polyvinylidene fluoride membrane (Biosharp, Wuhan, China) which was then blocked in blocking buffer, containing 5% skim milk powder, and was incubated with the primary antibodies of rabbit anti-SLC38A1 antibody (1:1000, ABclonal, Wuhan, China), rabbit anti-SLC38A2 antibody (1:1000, 159 BIOSS, Beijing, China), rabbit anti-β-casein antibody (1:500, ABclonal, Wuhan, China), rabbit anti-β-lactoglobulin antibody (1:500, BIOSS, Beijing, China), rabbit anti-PRLR antibody (1:1000, ABclonal, Wuhan, China), and β-actin mouse mAb (1:4000, AC004, ABclonal, Wuhan, China) at 4 °C overnight. After being washed in Tris-Buffered Saline and Tween 20 (TBST) 5 times, membranes were incubated with horseradish peroxidase (HRP)-conjugated secondary antibodies (Donkey anti-rabbit IgG or Donkey anti-mouse IgG, 1:4000, ABclonal, Wuhan, China). The target proteins were finally visualized with chemiluminescence ECL (Biosharp, Wuhan, China) and were quantified with Image-Pro plus 6.0 software (Media Cybernetics, Inc., Silver Spring, MD, USA). Protein levels were normalized to β-actin.

### 2.7. Dual-Luciferase Reporter Gene Assay

The promoters of *SNAT1* and *SNAT2* genes were truncated into three segments. The primers and the PCR assay for amplifying SNAT1/2 promoter segments are shown in Table S2. The truncated loci for SNAT1 promoters were −1724/+166, −1186/+166, and −629/+166 and for SNAT2 promoters were −1695/+95, −1353/+95, and −771/+95, respectively. These sequences were sub-cloned into pGL3 luciferase reporter vector which was kindly provided by Professor Wentao Li. The recombinant plasmids were designated as pGL3-SNAT1/2-1, pGL3-SNAT1/2-2, and pGL3-SNAT1/2-3 (Figure S1). For dual-luciferase reporter gene assay (Dual-Luciferase Reporter Assay System, Promega, Madison, WI, USA), cells with a density of 50% were transfected with pGL3-SNAT1/2-1, pGL3-SNAT1/2-2, and pGL3-SNAT1/2-3, together with the pRL-TK plasmid as an internal control using Lipofectamine 3000 (11668027, Invitrogen, Carlsbad, CA, USA). Cells transfected with the empty vector were used as negative controls. Then, cells were divided into four groups treated with 0 ng/mL, 20 ng/mL, 200 ng/mL, and 2000 ng/mL PRL, respectively, 24 h post-transfection. Cells were finally harvested for detection of luciferase activity 24 h after PRL administration.

### 2.8. RNA Interference

Short interfering RNAs (siRNAs) against SNAT1, SNAT2, PRLR, or STAT5 were purchased from GenePharma (Shanghai, China). Corresponding scrambled siRNAs were used as negative controls (NC groups). All siRNA sequences are shown in Table S3. Transient transfection was performed in cultured GMECs when the cell density reached a confluency of 70–80% using Lipofectamine 2000 reagent (Invitrogen, Waltham, MA USA). To knock down the target genes, cells were transfected with corresponding siRNAs. After 24 h transfection, cells were harvested for RNA extraction and qPCR analysis. After 48 h of transfection, cells were harvested for mRNA or protein extraction. 

### 2.9. Serum PRL Quantification

Blood was collected at 10:00 am from goats at puberty, Pd91, Pd137, Ld4, and Ld31. The blood sample was collected from the jugular vein of the goats. The serum was separated from the blood through incubation and centrifugation, respectively, and was stored at −80℃ before detection. Serum PRL was measured with goat PRL ELISA kit (Shanghai Bangyi Biotechnology Co., Ltd., Shanghai, China). For PRL immunoassay, the intra- and inter-assay coefficients of variation (CVs) were <15%. The detection limit for PRL was <1.0 ng/mL.

### 2.10. Cell Fatty Acids Quantification

The triglyceride (TG) in the cell supernatant was quantified with commercial kit (Nanjing Jiancheng Bioengineering Institute, Nanjing, China). For triglyceride determination, inter-assay coefficient of variation (CV) was <8%. The detection limit for TG was 0.3–11.4 mmol/L.

### 2.11. SNAT1 and SNAT2 Overexpression

The goat SNAT1 (XM_018047893.1) sequence was amplified from genomic DNA of goat mammary gland. The primers for amplifying SNAT1 were as follows: forward primer, 5′-tcagatctcgagctcaagctt CGATGATGCATTTCAAAAGTGGACTCGA -3′ (Hind III), and reverse primer, 5′-ttatctagatccggtggatcc TCAGTGGCCTTCGTCACCACT -3′ (BamH I). The amplified SNAT1 fragment was sub-cloned into an expression vector (pEGFP-C1) and using this company for sequencing (Genecreate, Wuhan, China). The recombinant plasmid was designated as pEGFP-SNAT1-C1 (Figure S2). The SNAT2 overexpression vector was constructed and verified in our previous study [27]. Cells were transfected with pEGFP-SNAT1-C1 or pEGFP-SNAT2-C1 (C1-SNAT1/2) using Lipofectamine 3000 (Invitrogen). Cells transfected with the empty vector were used as negative controls (C1 groups). Cells were harvested for mRNA and protein assays 24 h, 48 h, or 72 h post-transfection.

### 2.12. Transcriptome Analysis of GMECs Treated with PRL

According to previous qPCR results, the cells were divided into two groups to be treated with 0 ng/mL and 200 ng/mL PRL for 24 h, respectively, and were harvested for transcriptome analysis. The transcriptome data of SNAT1 and SNAT2 genes were analyzed with the assistance of company (Shanghai Majorbio Bio-pharm Technology Co., Ltd., Shanghai, China). Two groups of cells were preserved with trizol (AG21102, Accurate Biology, Changsha, China) and sent to the company for RNA extraction. The data on the difference maps for the genes were analyzed on the Majorbio Cloud Platform (www.majorbio.com, accessed on 15 January 2024). 

### 2.13. Statistical Analysis

Statistical analyses were performed using IBM SPSS Statistics 17.0 software (IBM, Armonk, New York, NY, USA). One-way analysis of variance was used to determine the differences among different groups. All data are presented as means ± SD. Statistical significance was declared when values of *p* < 0.05 or *p* < 0.01, which were indicated by different alphabetical letters or asterisks.

## 3. Results

### 3.1. Protein Expression of SNAT1/2 Are Up-Regulated in Goat Mammary Glands during Late Pregnancy and Early Lactation

**Figure 2:**
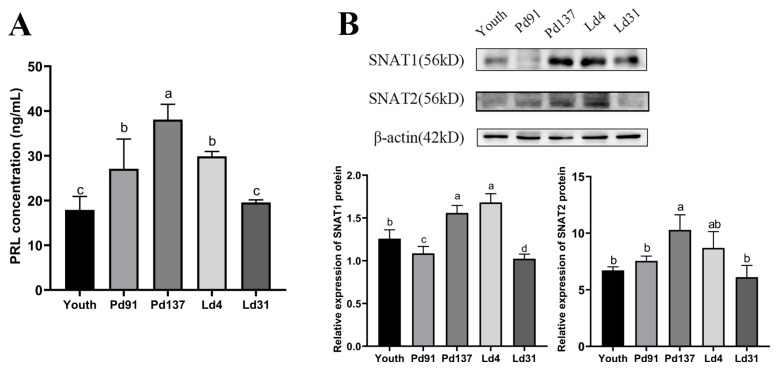
PRL and SNAT1/2 levels in different stages of goats. PRL: prolactin; SNAT1/2: sodium-coupled neutral amino acid transporter 1/2. (**A**) Serum PRL concentrations in goats. (**B**) SNAT1/2 protein expressions in different stages of goat mammary tissue. Youth: The goats are in the stage of puberty; Pd91: The goats are in the stage of pregnancy, day 91; Pd137: The goats are in the stage of pregnancy, day 137; Ld4: The goats are in the stage of lactation, day 4; Ld31: The goats are in the stage of lactation, day 31. Data are represented as means ± SD. Values with different lowercase letters indicate significant difference (*p* < 0.05). Original images can be found in Supplementary Files.

To determine whether levels of SNAT1 and SNAT2 are associated with PRL levels, we first detected serum PRL concentrations and mammary gland protein expression of SNAT1/2 in goats at the stages of puberty, Pd91, Pd137, Ld4, and Ld31 with ELISA and western blot analysis. We observed that PRL levels were higher on Pd137 or Ld4 than those on Pd91 (*p* = 0.01) or Ld31 (*p* = 0.005) (Figure 2A). Similarly, SNAT1 and SNAT2 proteins in goat mammary glands on Pd137 or Ld4 were higher than those on Pd91 (*p* < 0.001 and *p* < 0.001) or Ld31 (*p* < 0.001 and *p* < 0.001), respectively (Figure 2B). These results suggested the importance of SNAT1/2 and PRL on goat mammary gland development and milk lactation.

### 3.2. SNAT1/2 Proteins Are Up-Regulated by Specific Concentrations of PRL Which Promote the Proliferation of GMECs

**Figure 3:**
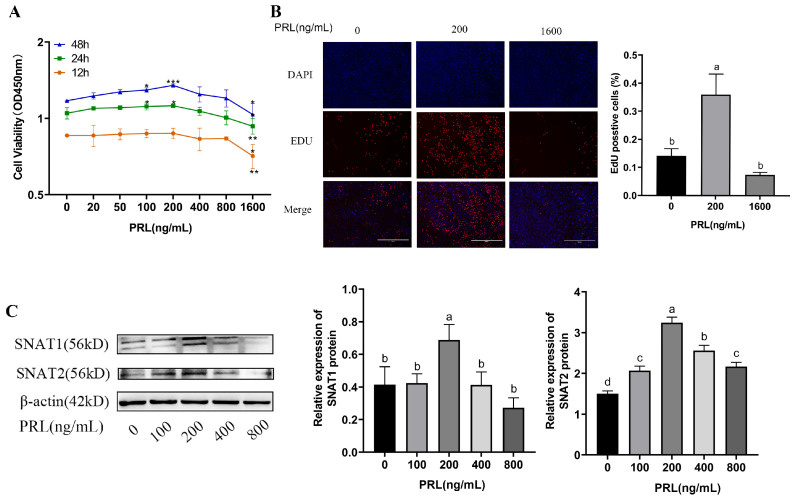
(**A**) GMECs are treated with different concentrations of PRL (0, 20, 50, 100, 200, 400, 800, and 1600 ng/mL) for 12, 24, and 48 h. Cell viability is determined by CCK-8 assay. (**B**) GMECs are treated with different concentrations of PRL (0, 200, and 1600 ng/mL) for 48 h. Cell viability is determined by EdU assay (400 μm). (**C**) Protein levels of SNAT1/2 in GMECs treated with PRL are detected by western blot analysis. Data are represented as means ± SD, * *p* < 0.05, ** *p* < 0.01, *** *p* < 0.001. Values with different lowercase letters indicate significant difference (*p* < 0.05). Original images can be found in Supplementary Files.

To determine whether SNAT1/2 expressions are related to PRL and the proliferation of GMECs, we treated GMECs with different concentrations of PRL for 12, 24, and 48 h. As shown in Figure 3A, a CCK-8 assay showed that the proliferation of GMECs was gradually elevated when the PRL concentration increased from 0 ng/mL to 200 ng/mL 24 h or 48 h after PRL administration on GMECs. To corroborate that 200 ng/mL PRL promoted the proliferation of GMECs best, we used a EdU assay to detect the proliferation of GMECs after being stimulated with PRL for 48 h. The largest (*p* = 0.001) number of EdU-positive cells occurred in the 200 ng/mL PRL group (Figure 3B). The protein expressions of SNAT1 and SNAT2 were also demonstrated in vitro 48 h after PRL stimulation. Western blot assays showed that the protein levels of SNAT1 and SNAT2 were gradually elevated and peaked when the PRL concentration increased from 0 to 200 ng/mL (Figure 3C). These results corroborate that SNAT1/2 played an important role in the regulation of PRL on GMECs’ proliferation.

### 3.3. SNAT1/2 Proteins Are Up-Regulated by Specific Concentrations of PRL Which Promote the Lactation of GMECs

**Figure 4:**
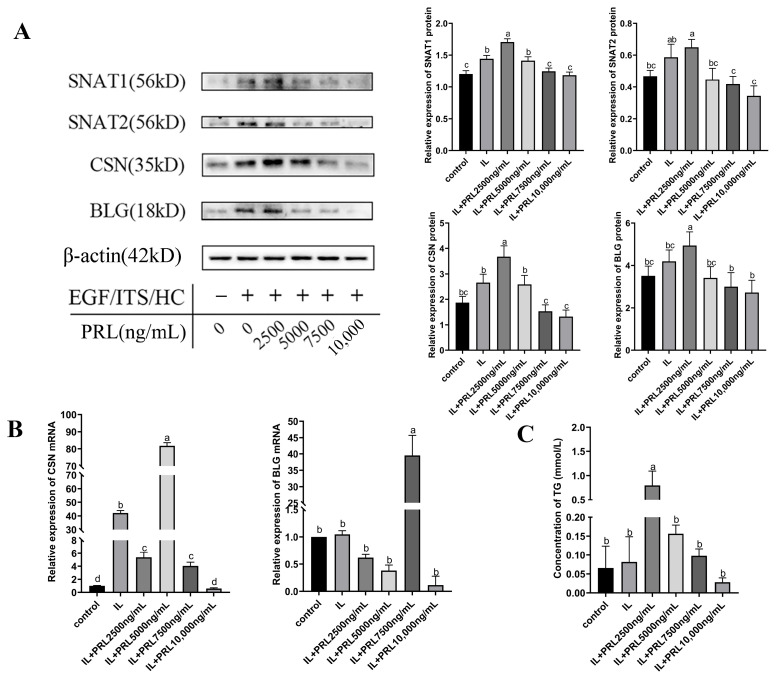
The effect of PRL on GMECs’ lactation and SNAT1/2 expression. (**A**) Western blot analysis of milk protein and SNAT1/2 in GMECs treated with PRL. (**B**) QPCR analysis of *CSN* and *BLG* mRNA levels in GMECs treated with PRL. *CSN*: Casein; *BLG*: Beta-lactoglobulin. (**C**) GPO-PAP assay of TG content in the supernatant of GMECs after induction of lactation. EGF: epidermal growth factor; ITS: insulin-transferrin-selenium; HC: hydrocortisone; Control: GMECs are treated with only culture medium; IL: GMECs are treated with an induced lactation system (EGF + ITS + HC); IL + PRL2500/5000/7500/10,000 ng/mL: GMECs are treated with IL and 2500, 5000, 7500, or 10,000 ng/mL PRL. Data are represented as means ± SD. Values with different lowercase letters indicate significant difference (*p* < 0.05). Original images can be found in Supplementary Files.

The levels of milk protein and fat indicate the nutritional value of milk. The relationship between SNAT1/2 and PRL-induced lactation was also demonstrated in GMECs. Under the inductive lactation system (10 ng/mL EGF, 1×ITS, and 1 μg/mL hydrocortisone), GMECs were treated with different concentrations of PRL for 48 h. A western blot assay showed that β-casein (*CSN*) and β-lactoglobulin (*BLG)* in GMECs were gradually elevated and then reached a peak, respectively, when treated with PRL from 0 ng/mL to 2500 ng/mL, which corresponded to the increase of SNAT1/2 proteins (Figure 4A). QPCR experiments detecting mRNA levels of milk proteins revealed that the mRNA levels of *CSN* and *BLG* increased, respectively, when treated with 5000 ng/mL or 7500 ng/mL PRL (Figure 4B). Additionally, a GPO-PAP (Phosphoglycerol oxidase-peroxidase coupling method) assay showed a similar change in TG content which also reached a peak when treated with 2500 ng/mL PRL (Figure 4C). These results indicated that SNAT1 and SNAT2 were involved in PRL-induced lactation in GMECs.

### 3.4. SNAT1/2 Are Involved in PRL Modulation of the Proliferation and Lactation of GMECs

**Figure 5:**
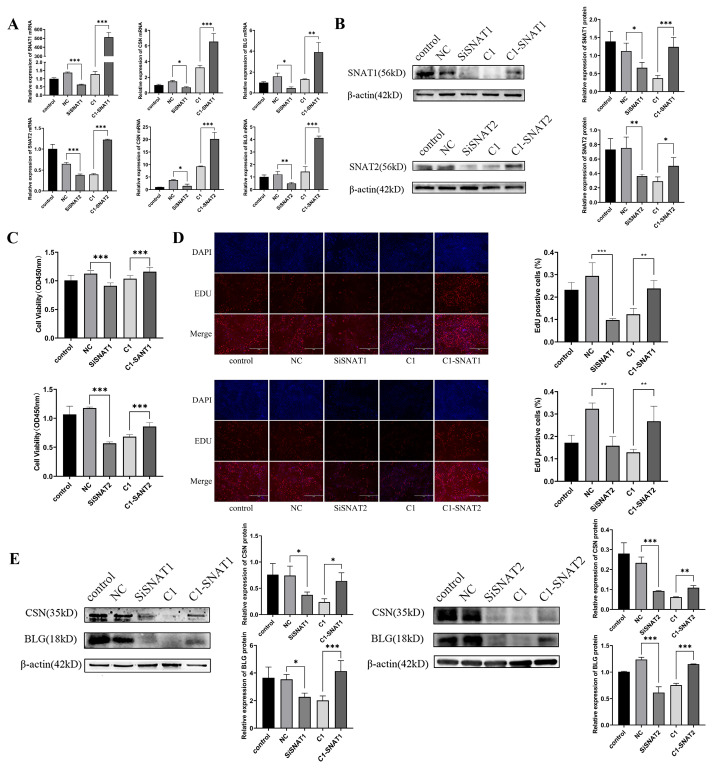
The effect of knockdown and overexpression of SNAT1/2 on GMECs’ proliferation and lactation. (**A**) The effect of knockdown and overexpression of SNAT1 or SNAT2 in GMECs on mRNA levels of *SNAT1/2*, *CSN*, and *BLG*. (**B**) Protein changes of SNAT1 and SNAT2 after they are knocked down or are overexpressed in GMECs. (**C**) Cell proliferative activity is detected by CCK-8 assay after knockdown or overexpression of SNAT1/2. (**D**) Cell proliferation activity is detected by EdU assay after knockdown or overexpression of SNAT1/2 (400 μm). (**E**) CSN and BLG protein changes in GMECs after being knocked down or overexpressed SNAT1/2. Control: GMECs did not receive any treatment; NC: GMECs transfected with corresponding scrambled siRNA for SNAT1 or SNAT2 as negative control; SiSNAT1/2: GMECs are transfected with siRNAs for SNAT1/2; C1: pEGFP-C1, GMECs are transfected with pEGFP-C1 empty vector (without a SNAT1 or SNAT2 sequence insert); C1-SNAT1/2: pEGFP-SNAT1/2-C1, GMECs are transfected with an overexpression vector for SNAT1/2. Data are represented as means ± SD, * *p* < 0.05, ** *p* < 0.01, *** *p* < 0.001. Original images can be found in Supplementary Files.

To demonstrate whether SNAT1/2 are involved in the modulation of PRL on GMECs’ proliferation and lactation, we knocked down and overexpressed SNAT1/2 expression by transfecting GMECs with siRNAs against SNAT1/2 mRNAs or SNAT1/2 overexpression vectors (Figure 5A,B). The CCK-8 assay showed that the cell viability was inhibited after *SNAT1/2* mRNAs interfered with siRNAs (SiSNAT1 vs. NC, *p* < 0.001; SiSNAT2 vs. NC, *p* < 0.001). In contrast, the cell viability was enhanced after SNAT1/2 were overexpressed (SNAT1, C1 vs. C1-SNAT1, *p* = 0.002; SNAT2, C1 vs. C1-SNAT2, *p* < 0.001) (Figure 5C). A EdU assay further demonstrated that the number of EdU-positive cells was reduced (SiSNAT1 vs. NC, *p* < 0.001; SiSNAT2 vs. NC, *p* = 0.001) or was elevated (SNAT1, C1 vs. C1-SNAT1, *p* = 0.002; SNAT2, C1 vs. C1-SNAT2, *p* = 0.002) when compared with those in the negative control group after SNAT1/2 were inhibited or enhanced in GMECs (Figure 5D). The decrease and increase in CSN and BLG levels after SNAT1/2 being knocked down or overexpressed, respectively, as assessed by western blot analysis, demonstrated the involvement of SNAT1/2 in GMECs’ lactation (Figure 5E). These data showed that SNAT1/2 played vital roles in PRL modulation on GMECs’ proliferation and lactation. 

### 3.5. SNAT1/2 mRNAs Are Up-Regulated in Response to PRL Stimulation in GMECs

**Figure 6:**
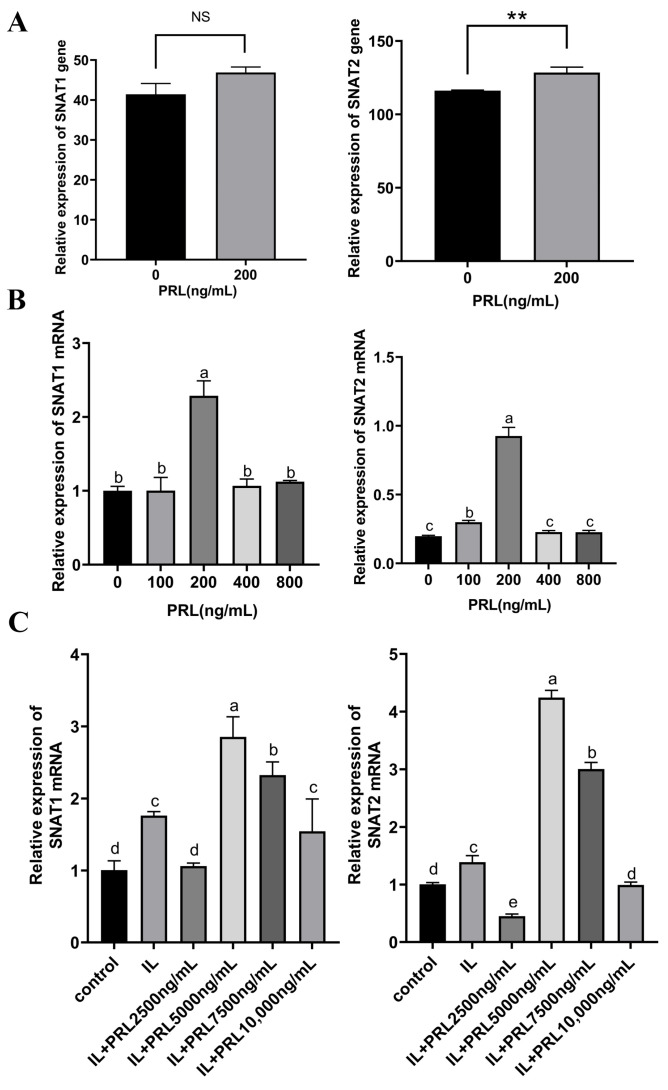
SNAT1/2 nucleic acid level detection. (**A**) RNA levels of *SNAT1/2* are detected by transcriptome sequencing after treatment with 200 ng/mL of PRL. (**B**) qPCR assays of *SNAT1/2* mRNA levels in GMECs after being treated with PRL (0, 100, 200, 400, 800 ng/mL). (**C**) qPCR assays for mRNA levels of *SNAT1/2* in GMECs after being treated with induced lactation system (EGF + ITS + HC) supplemented with PRL (0, 2500, 5000, 7500, 10,000 ng/mL). EGF: epidermal growth factor; ITS: insulin-transferrin-selenium; HC: hydrocortisone; IL: GMECs are treated with an induced lactation system with EGF, ITS, and HC; IL + PRL2500/5000/7500/10,000 ng/mL: GMECs are treated with IL and 2500, 5000, 7500, or 10,000 ng/mL PRL. Data are represented as means ± SD, NS: no statistical significance, ** *p* < 0.01. Values with different lowercase letters indicate significant difference (*p* < 0.05).

Since SNAT1/2 have been verified to be involved in the regulation of PRL in the proliferation and lactation of GMECs, the next step was to clarify whether PRL regulates the expression of SNAT1/2. As shown in Figure 6A, transcriptome RNA sequencing showed that the highest mRNA expression of *SNAT1/2* occurred in 200 ng/mL PRL, which was also confirmed by qPCR assays (Figure 6B,C). Since SNAT1 and SNAT2 are coded by *SLC38A1* and *SLC38A2* genes, the results illustrated that PRL may affect the transcriptional activation of *SLC38A1* and *SLC38A2*.

### 3.6. Promoter Regions of SNAT1 and SNAT2 Are Activated by PRL

**Figure 7:**
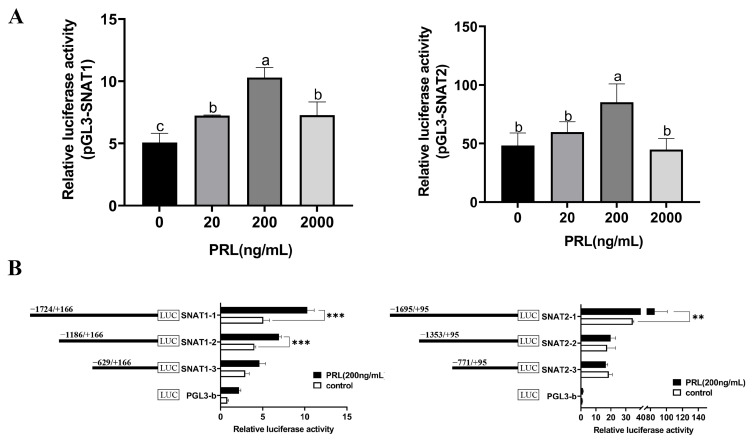
The effect of PRL on SNAT1/2 transcription initiation. (**A**) Dual-luciferase reporter gene assays on SNAT1/2 gene transcriptional activity in GMECs treated with PRL. (**B**) Dual-luciferase reporter gene assays of truncated SNAT1/2 promoter activity in GMECs treated with PRL. pGL3-SNAT1/2: pGL3 vectors containing the SNAT1/2 promoter sequence (−1724/+166)/(−1695/+95); PRL (200 ng/mL): GMECs are treated with 200 ng/mL prolactin; Control: GMECs did not receive any treatment; SNAT1-1/2/3: The SNAT1 promoter sequence is truncated into three segments, which are respectively inserted into pGL3 vectors; SNAT2-1/2/3: The SNAT2 promoter sequence is truncated into three segments, which are respectively inserted into pGL3 vectors; pGL3-b: GMECs are transfected into pGL3-basic vector. Data are represented as Means ± SD, ** *p* < 0.01, *** *p* < 0.001. Values with different lowercase letters indicate significant difference (*p* < 0.05).

To determine whether promoter regions of SNAT1/2 could be activated by PRL, we performed luciferase reporter gene assays. We observed (Figure 7A) that the highest promoter activity occurred when 200 ng/mL PRL was administered 24 h after stimulation in GMECs. Next, we observed that 200 ng/mL PRL increased promoter activity by approximately 2.03 times when using base pairs −1724 to +166 of the SNAT1 promoter region and 1.77 times when using base pairs −1695 to +95 of the SNAT2 promoter region (Figure 7B). In order to identify the detailed promoter region of SNAT1/2 that exerts the main function, we used a series of unidirectional 5′ deletion constructs generated from the complete sequence. The PRL-induced promoter activities were significantly reduced with the constructs encoding base pairs −1186 to +166 and −629 to +166, respectively, of SNAT1, and −1353 to +95 and −771 to +95, respectively, of SNAT2. Deletion of base pairs −1724 to −629 of SNAT1 and −1695 to −1353 of SNAT2, respectively, resulted in the loss of PRL-induced activity. 

### 3.7. PRL Activates SNAT1/2 through the PRLR-STAT5-Dependent Pathway

**Figure 8:**
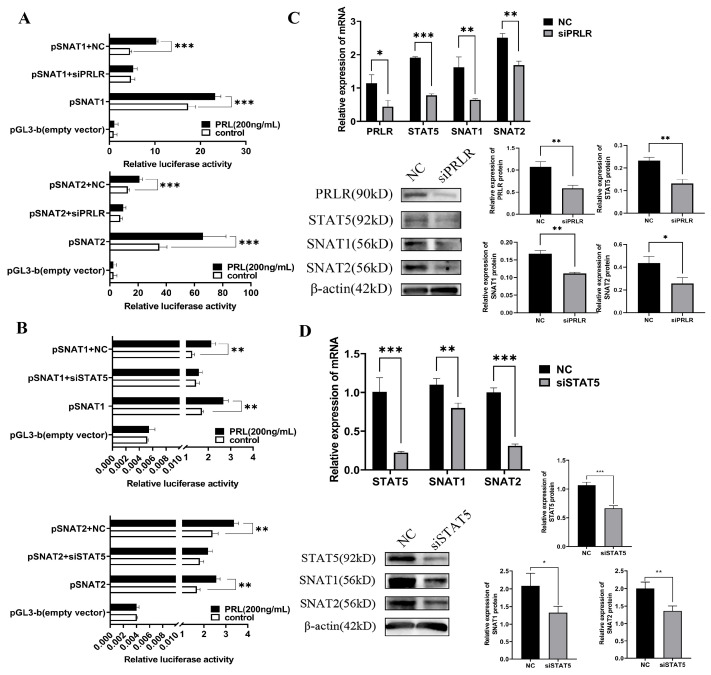
The effect of PRLR-STAT5 on transcriptional activity of SNAT1/2 regulated by PRL. PRLR: prolactin receptor; STAT5: signal transducers and activators of transcription 5. (**A**) SNAT1/2 promoter fluorescence activity is measured after PRLR is knocked down. (**B**) Promoter fluorescence activity of SNAT1/2 in GMECs after knocked down STAT5. (**C**) Protein and mRNA levels of STAT5 and SNAT1/2 are measured after knockdown of PRLR. (**D**) Protein and mRNA levels of SNAT1/2 in GMECs after knocked down STAT5. pSNAT1/2-NC: negative control for PRLR/STAT5 siRNAs during SNAT1/2 promoter assay; pSNAT1/2 + siPRLR/siSTAT5: The SNAT1/2 promoter–reporter assay was accompanied by PRLR or STAT5 RNA interference; pSNAT1/2: The SNAT1/2 promoter–reporter assay accompanied with pGL3-basic vector; NC: the negative control for PRLR or STAT5 RNA interference; siPRLR/siSTAT5: siRNAs for PRLR or STAT5. Data are represented as means ± SD, * *p* < 0.05, ** *p* < 0.01, *** *p* < 0.001. Original images can be found in Supplementary Files.

Having confirmed the functional role of PRL in the activation of SNAT1/2 promoters in GMECs, we next attempted to understand the mechanism underlying the transient up-regulation of SNAT1/2 in response to PRL stimulation. Previous evidence indicated that PRL promoted cell proliferation and milk lactation through PRLR and STAT5 signaling during the lactation period, and STAT5 is an important downstream signaling factor of PRLR [4,28,29]. We hypothesized that the rapid induction of SNAT1/2 in response to PRL might depend on the PRLR-STAT5 pathway. Therefore, we examined the hypothesis in vitro in GMECs. Accompanied by rapidly decreased PRLR (Figure 8A) or STAT5 levels (Figure 8B) interfered with by siRNAs of PRLR or STAT5, respectively, in GMECs, we observed the loss of PRL-induced luciferase activity in SNAT1 or SNAT2 promoters. Furthermore, qPCR and western blotting assays showed a marked decrease in STAT5, SNAT1, and SNAT2 mRNAs and proteins after PRLR or STAT5 was knocked down by their siRNAs (Figure 8C,D). Thus, these results indicated that the PRLR-STAT5 pathway was essential for PRL to activate the transcriptional system of SNAT1 and SNAT2. However, we did not observe the direct binding of SNAT5 with promoter regions of SNAT1/2 by electrophoretic mobility shift assay (EMSA) (Figure S3). Collectively, these results demonstrated that PRL induction of transcriptional activation of SNAT1/2 was mediated by PRLR-STAT5 signaling.

## 4. Discussion

The crossbreeding of Boer goats and Macheng black goats is prompted in Hubei Province. Clarifying the mechanisms of hormone regulation on goat mammary glands in this goat breed is beneficial for animal reproduction and local economics. The primary hypothesis of our study is that SNAT1/2 mediate the effects of PRL on GMECs’ proliferation and lactation. We further hypothesize that PRL regulates the transcription and expression of SNAT1 and SNAT2 genes through the PRLR-STAT5 signaling pathway. The current study demonstrated that PRL activated both the transcriptional expressions of SNAT1 and SNAT2 via PRLR-STAT5 signaling to promote the proliferation and secretion of goat mammary epithelial cells. These findings yield new insights into the regulatory mechanisms of endocrines on ruminant mammary growth and milk yields.

Gestation and lactation are two specific stages for mammals; during these two stages, the animals initiate a complex endocrine program to prepare the mammary gland to be a secretory organ for feeding the new-born animals [30,31,32].Therefore, a few genes are turned on for the proliferation and secretion of the mammary gland during these two periods for the synthesis of nutrients for the newborn [33,34,35].To trigger this complex process, the hormonal environment during these stages controls the transcription of related genes [36]. SNAT1 and SNAT2 are two main amino acid transporters in system A that enhance the uptake of vital amino acids such as alanine and glutamine into cells. Our results showed that SNAT1/2’s protein expression is up-regulated in goat mammary glands during late pregnancy and early lactation, consistent with the changes in serum PRL levels. This suggests that hormonal changes promote SNAT1/2 expression during these physiological stages to meet the increased nutritional requirements. Another study has demonstrated that 17β-estradiol interacts with ER-α to promote the transactivation of the SNAT2 gene through the ERE element during late pregnancy and early lactation [27,37], indicating that SNAT1/2 are crucial transporters, mediating hormones to stimulate the mammary function. Researchers also found that PRL regulated the expression of SNAT2 in rat mammary explants by increasing both the mRNA and protein levels of SNAT2 [24]. Also, a decrease in fetal weight has also been visualized due to the inhibition of system A by an amino acid analog during gestation, indicating the insufficiency of the mammary secretion [38]. In accordance with this, the higher protein expression of SNAT1 and SNAT2 during late gestation and early lactation indicated elevated demand for the SNAT1- and SNAT2-transported amino acids in the goat mammary gland.

In particular, PRL has been demonstrated to be a powerful activator of genes involved in cell proliferation [39], differentiation, and secretion in ruminants [40,41]. In the current study, we used the concentrations of 20 to 1000 ng/mL PRL to evaluate the effect of PRL on cell proliferation. These concentrations contain the broad range of in vivo serum physiological levels of PRL. However, due to factors such as cell membrane permeability and receptor availability, the in vitro experiments in cells may typically require higher concentrations than those in vivo to elicit a measurable response. This may explain why 200 ng/mL PRL promoted cell viability the most. However, 1000 ng/mL PRL decreased cell viability; this may illustrate that 1000 ng/mL PRL is harmful to cell growth (induces cell apoptosis or stress), suggesting a dose-dependent effect of PRL on GMECs. Some other studies also used these concentrations to promote cell proliferation [42,43]. Meanwhile, we used the concentrations of 2500–10,000 ng/mL PRL since we wanted to induce the lactation of GMECs which may secrete milk proteins and fat acids. The conventional lactation induction system which contains insulin, cortisol, epithelial growth factor, and PRL is controlled in a condition where the cells reach at least 80% confluency and no longer proliferate. In this conventional lactation induction system, 5000 ng/mL PRL was commonly used to induce ruminant mammary cell lactation [44,45]. We used several concentrations in the induction system since we wanted to maximize the PRL biological effect, and we found that the optimal concentration of PRL to induce the expression of SNATs was 2500 ng/mL. However, we may see protein expression in GMECs administered with and without PRL in Figure 4A. This may be due to GMECs having a basal secretory activity since the mammary gland itself could synthesize hormones to stimulate secretion, which leads to the detectable protein expression even without PRL or other factors [46]. However, when added to the lactation induction system containing EGF, insulin, and hydrocortisone without PRL, the protein expression such as SNATs and milk proteins increased, indicating that these factors can promote cell secretion. When PRL and the induction factors were both added to the GMECs, the proteins’ expression in GMECs was higher than those groups with induction factors alone, suggesting that PRL played a crucial role in inducing and maintaining the lactation mammary secretion. Furthermore, the SNAT overexpression (Figure 5B,E) levels were below the non-treated controls but were higher than the control plasmid-treated cells. This may be due to the toxicity of transfection reagents and the large size of the overexpression plasmids [47,48]. These factors inhibited the cell viability and increased the cellular burden after transfection, thus leading to a great decrease in overall protein expression in the SNAT overexpression group. But the higher expression of SNATs in the overexpression group than those in the control plasmid group demonstrated that the SNATs were overexpressed. Similar problems also occurred in siSNAT transfections. The knockdown and overexpression of SNAT1/2 verified our hypothesis that SNAT1/2 are involved in the modulation of PRL on the proliferation and secretion of GMECs. These results agree with previous findings that SNAT2 is involved in cell proliferation [15,49] and mediates the regulation of PRL in cow or rat mammary cell or tissue secretion [23,24]. Our results further suggest that PRL modulates the proliferation and secretion of GMECs through enhancing the uptake of SNAT1/2-transported amino acids.

Transcriptome RNA sequencing data and the subsequent qPCR analysis indicated that PRL affected the GMECs’ physiological process through activating *SNAT1/2* gene transcription. The luciferase reporter gene assays further verified the SNAT1/2 promoter regions (SNAT1: −1724 to −629 bp; SNAT2: −1695 to −1353 bp) were stimulated by PRL. STAT5 can be activated by hormones (e.g., PRL, growth hormone), cytokines (e.g., IL-2, IL-3, IL-5), and other kinases [50,51,52]. These factors can phosphorylate STAT5 and further promote STAT5 activation. PRL usually regulated the downstream genes via the JAK-STAT5 signaling pathway [53,54,55]. Researchers have found that PRL enhanced milk protein synthesis in mammary epithelial cells of dairy cows by modulating LAT1 expression via the STAT5 signaling pathway [3]. Furthermore, the function of PRL on the cells takes place via its receptor (PRLR) which activates JAK2 and, subsequently, STAT5 to translocate into the nucleus and then bind to the regulatory regions of target genes to initiate transcription [56,57,58]. Commonly, STAT5 directly binds to the gene promoter region to initiate gene transcription [59]. However, sometimes STAT5 interacts with various genomic regions including enhancers and other regulatory elements beyond promoters [60,61]. In addition, STAT5 can also regulate gene expression by recruiting chromatin remodeling complexes to change the state of chromatin and thus promote gene transcription [62]. Some other studies found that STAT5 can regulate the downstream non-coding RNA of genes or affect other signaling pathways to modulate target genes [63,64]. In the current study, PRLR-STAT5 mediates PRL-induced transcriptional activation of SNAT1 and SNAT2. However, we did not observe the direct binding of STAT5 with the promoter regions of SNAT1/2 by EMSA (Figure S3), indicating that STAT5 may co-localize with the enhancers or other 5′ flanking regions of SNAT1/2 genes or affect other factors to regulate SNAT1/2 expression. These complex regulatory mechanisms may explain why STAT5 binding is not observed at the SNAT1/2 promoter, but its regulatory role remains evident.

## 5. Conclusions

This study demonstrates that prolactin (PRL) regulates the expression of the amino acid transporters SNAT1 and SNAT2 in goat mammary epithelial cells in vivo and in vitro, and that the PRLR-STAT5 signaling pathway is essential for PRL-induced transcriptional activation of these amino acid transporters. In addition, the similar function of SNAT1 and SNAT2 suggests that the family of system A amino acid transporters played a similar role in participating in PRL regulation of mammary function. These findings provide insights into how PRL affects mammary gland development and lactation in ruminants by regulating amino acid transporter expression.

## Data Availability

All data generated or analyzed during this study are included in this published article.

## Supplementary Materials

The following supporting information can be downloaded at: https://www.mdpi.com/article/10.3390/cells13171461/s1. Figure S1: Schematic representation of SNAT1 and SNAT2, each truncated into three segments and inserted into the pGL3 vector; Figure S2: Plasmid profile of the pEGFP-SNAT1-C1 overexpression vector and its multiple cloning site; Figure S3: Electrophoretic Mobility Shift Assay (EMSA) analysis of STAT5 binding to the SNAT1/2 promoter. SNAT1/2-labeled probes were incubated separately with a nuclear extract, while the labeled probe without a nuclear extract served as a negative control; Table S1: Primer sequences used for quantitative real-time PCR; Table S2: Primers for the amplification of SNAT1/2 promoters and deletion constructs; Table S3: The sequences of siRNAs for NC, SNAT1, SNAT2, PRLR, and STAT5. Original images can be found in Supplementary Files.
