# Supplementary material for: Prolactin Modulates the Proliferation and Secretion of Goat Mammary Epithelial Cells via Regulating Sodium-Coupled Neutral Amino Acid Transporter 1 and 2
Source: Cells. 2024 Aug 30;13(17):1461. doi: 10.3390/cells13171461 (PMC11394342; doi:10.3390/cells13171461)
Supplement: Supplementary file 1 [file cells-13-01461-s001.zip › SUPPLEMENTAL INFORMATION/SUPPLEMENTAL INFORMATION.pdf]

SUPPLEMENTAL INFORMATION for

Prolactin Modulates the Proliferation and Secretion of Goat Mammary Epithelial Cells via regulating Sodium-Coupled Neutral Amino Acid Transporter 1 and 2

Figures

Figure S1

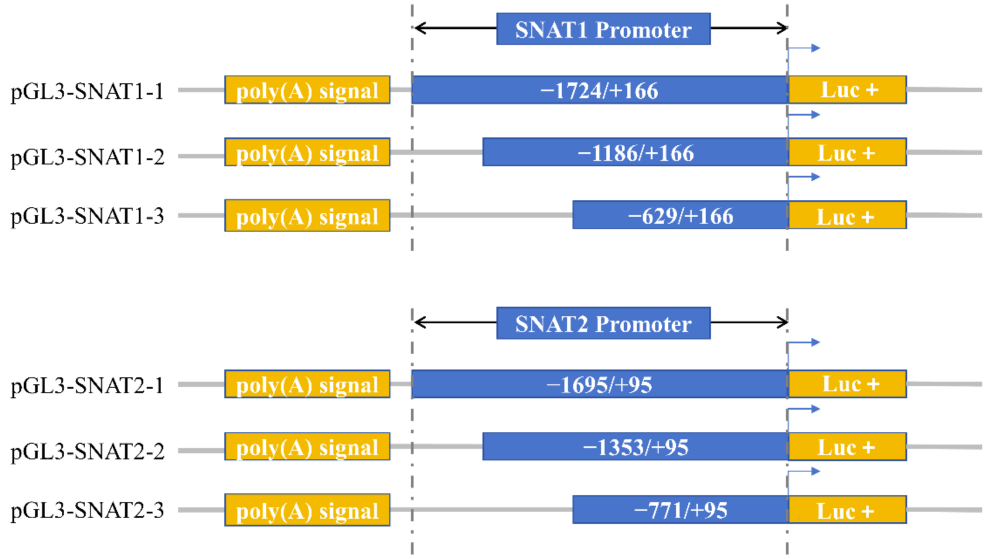

Figure S1. Schematic representation of SNAT1 and SNAT2, each truncated into three segments and inserted into the pGL3 vector.

Figure S2

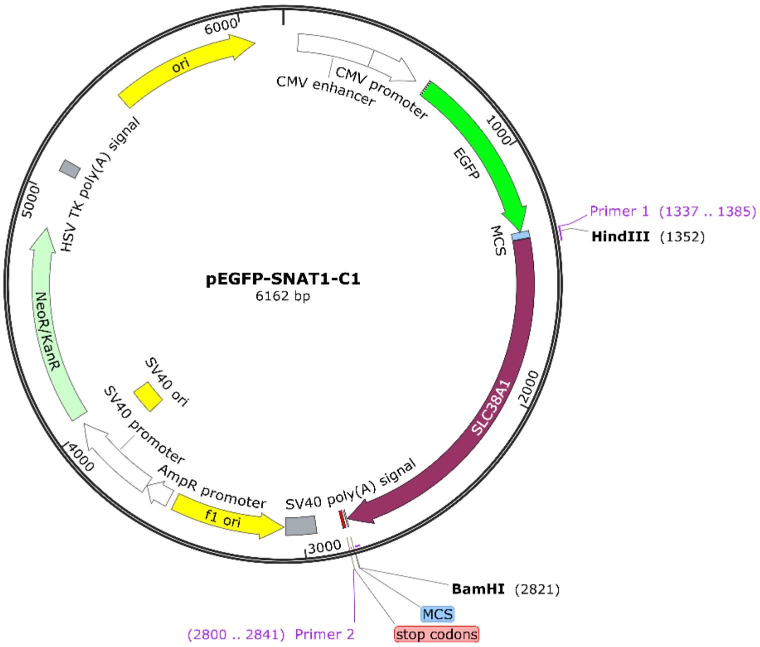

Figure S2. Plasmid profile of the pEGFP-SNAT1-C1 overexpression vector and its multiple cloning site.

Figure S3

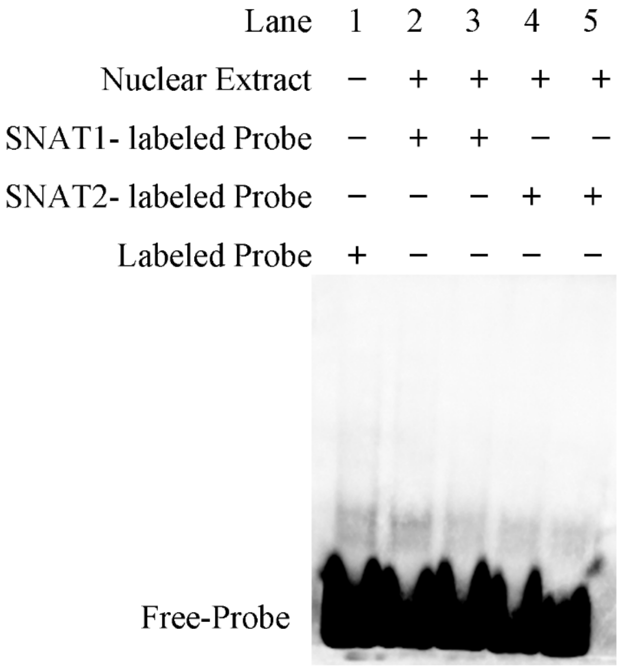

Figure S3. Electrophoretic Mobility Shift Assay (EMSA) analysis of STAT5 binding to the SNAT1/2 promoter. SNAT1/2- labeled probes were incubated separately with a nuclear extract, while the labeled probe without a nuclear extract served as a negative control. Lane: samples loaded in each lane, 1: labeled probe without GMECs nuclear extract, 2/ 3: SNAT1-labeled probe with GMECs nuclear extract, 4/5: SNAT2- labeled probe with GMECs nuclear extract.

EMSA protocol

According to the manufacturer's protocol, specific biotin-labeled probes targeting SNAT1 and SNAT2 were prepared using reagents from Accurate Biology (Changsha, China). The nuclear extracts from GMECs were obtained using the Nuclear and Cytoplasmic Protein Extraction Kit (Beyotime, Shanghai, China). The EMSA kit (Beyotime) was used to detect the binding between nuclear extracts from GMECs and SNAT1/2 labeled probes. Subsequently, the biotin-labeled SNAT1/2 probes were incubated with or without GMECs nuclear extracts in EMSA/Gel-Shift binding buffers. The incubation was carried out at room temperature for 20 min. The binding reactions were resolved on a 6% non-denaturing PAGE gel at 100 V for 1 h. Following electrophoresis, the gel was subjected to wet electro-transfer at 380 mA for 1 h to transfer the binding and free probes onto a nylon membrane. The membrane was then cross-linked under ultraviolet light for 10 min. After cross-linking, the nylon membrane was blocked with Streptavidin-HRP conjugate blocking solution for 15 min. The membrane was subsequently washed three times in washing buffer, and the probes were visualized using BeyoECL Moon reagent according to the manufacturer's instructions.

Tables

Table S1. Primer sequences used for quantitative real-time PCR

Gene	GenBank accession number	Primer sequence (5’ to 3’)	bp
SNAT1	XM_018047893.1	F: TTAGGTTATCTTGGCTATACGA<br>R: TGTTGAATTAGGACTTGTTGAA	132
SNAT2	XM_018047891.1	F: CAATAAACGGCAGCTTAACACAC<br>R:GCAAAAAAGGAAATCTTGGACAC	235
CSN	NM_001009373.1	F:AATGTAGTCGGTGAGACTGTGGAA<br>R:TGAGTAAGAGGCAGGATGTTTTGT	218
BLG	X04520.1	F: GATCCCTGCGGTGTTCAAG<br>R: TCGGGCTCAGCACTGTTTT	111
PRLR	NM_001285669.1	F: TGAACCAGAGCCTCCTATGAACC<br>R: AAATGAGTCTCCCAGTCAGTTGC	177
STAT5	XM_018065115.1	F: AGATGCTGGCTGAGGTCAAC<br>R: GCTGCTTCTCGATGATGAATGTG	81
β-actin	NM_001009784.3	F: GGATGATGATATTGCTGCGCTC<br>R: TCTCCATGTCGTCCCAGTTGG	248

Table S2. Primers for the amplification of SNAT1/2 promoters and deletion constructs

Gene	Truncated loci	Primer sequence (5' to 3')	bp
SNAT1-1	−1724/+166	F:ATTTCTCTATCGATAGGTACCACCAGCTGATGGAGCCATGCT<br>R:CACGCGTAAGAGCTCGGTACCTACCCACACGGTACTCGCCA	1890
SNAT1-2	−1186/+166	F:ATTTCTCTATCGATAGGTACCTCCATCAGAGGCTACTCCGTCT<br>R:CACGCGTAAGAGCTCGGTACCTACCCACACGGTACTCGCCA	1352
SNAT1-3	−629/+166	F:ATTTCTCTATCGATAGGTACCGGACAAAGGCTTTCCAGAGGT<br>R:CACGCGTAAGAGCTCGGTACCTACCCACACGGTACTCGCCA	795
SNAT2-1	−1695/+95	F:ATTTCTCTATCGATAGGTACCAGTCACTCAGTCATGTTCTACTCC<br>R:CACGCGTAAGAGCTCGGTACCTGTTACCTTGGTGGTCTGGGCTC	2250
SNAT2-2	−1353/+95	F:ATTTCTCTATCGATAGGTACCACAGCCCTTGCAATAAGAGGA<br>R:CACGCGTAAGAGCTCGGTACCTGTTACCTTGGTGGTCTGGGCTC	1633
SNAT2-3	−771/+95	F:ATTTCTCTATCGATAGGTACCTGAGCGAAACGTGACCAGGA<br>R:CACGCGTAAGAGCTCGGTACCTGTTACCTTGGTGGTCTGGGCTC	1051

Note: PCR amplification conditions were as follows: initial denaturation at 94°C for 3 minutes, 35 cycles of 98°C for 10 seconds, 55°C for 30 seconds, 72°C for 1 minute.

Table S3. The sequences of siRNAs for NC, SNAT1, SNAT2, PRLR, and STAT5

SiRNA	Primer sequence (5' to 3')
SiRNA-NC	F：UUCUCCGAACGUGUCACGUTT
	R：ACGUGACACGUUCGGAGAATT
SiSNAT1	F：GCCCGAAGAUGAUAACAUUTT
	R：AAUGUUAUCAUCUUCGGGCTT
SiSNAT2	F：GCUCUGUUCUUCCUGCUAATT
	R：UUAGCAGGAAGAACAGAGCTT
SiPRLR	F：GGACCCAAAUCUUCCACAUTT
	R：AUGUGGAAGAUUUGGGUCCTT
SiSTAT5	F：GCAGCAGACUCAAGAGUAUTT
	R：AUACUCUUGAGUCUGCUGCTT
