# Supplementary figures and images for: Prolactin Modulates the Proliferation and Secretion of Goat Mammary Epithelial Cells via Regulating Sodium-Coupled Neutral Amino Acid Transporter 1 and 2
Source: Cells. 2024 Aug 30;13(17):1461. doi: 10.3390/cells13171461 (PMC11394342; doi:10.3390/cells13171461)

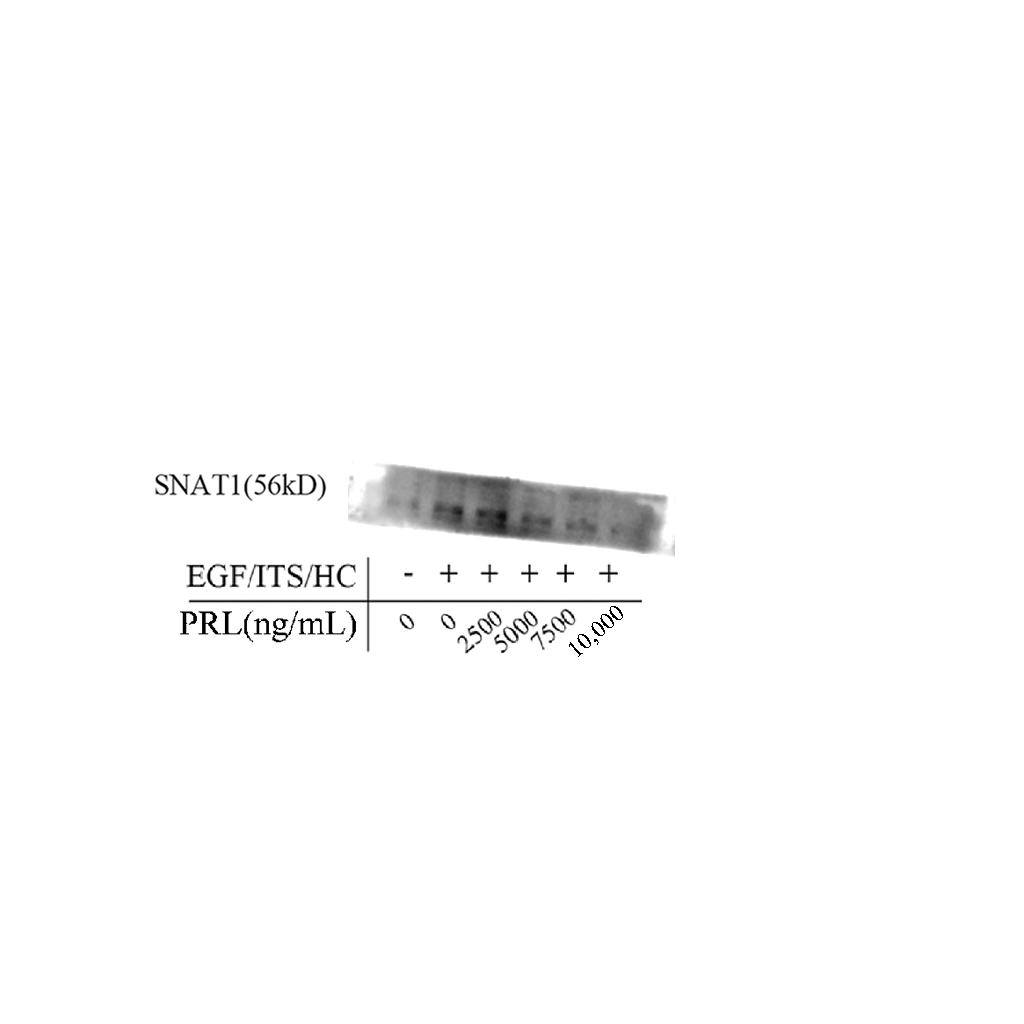

Supplement: Supplementary file 1 [file cells-13-01461-s001.zip › Original Images for Blots/Figure 4A/SNAT1.Tif]

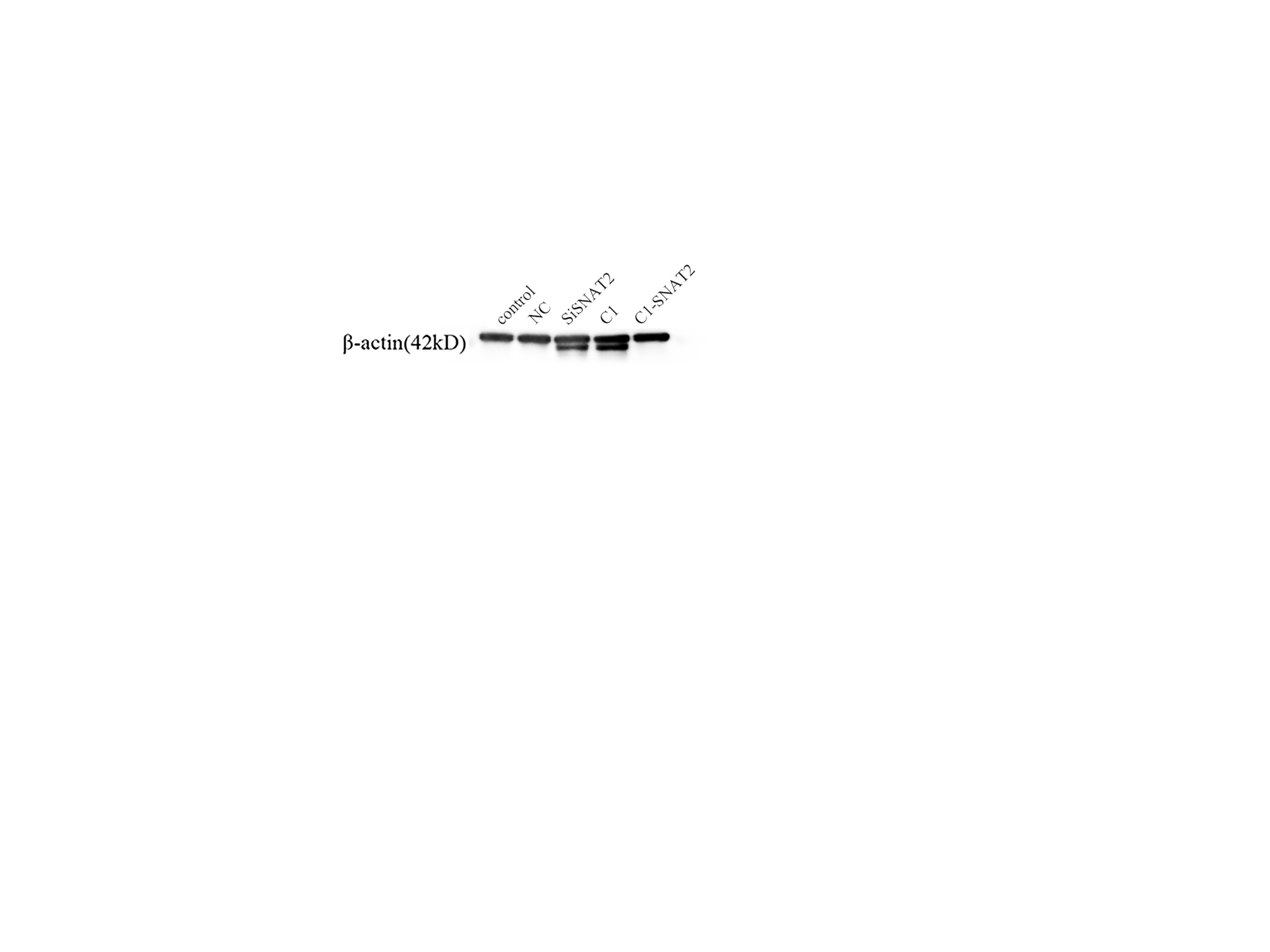

Supplement: Supplementary file 1 [file cells-13-01461-s001.zip › Original Images for Blots/Figure 5E/b-actn(SiSNAT2).tif]

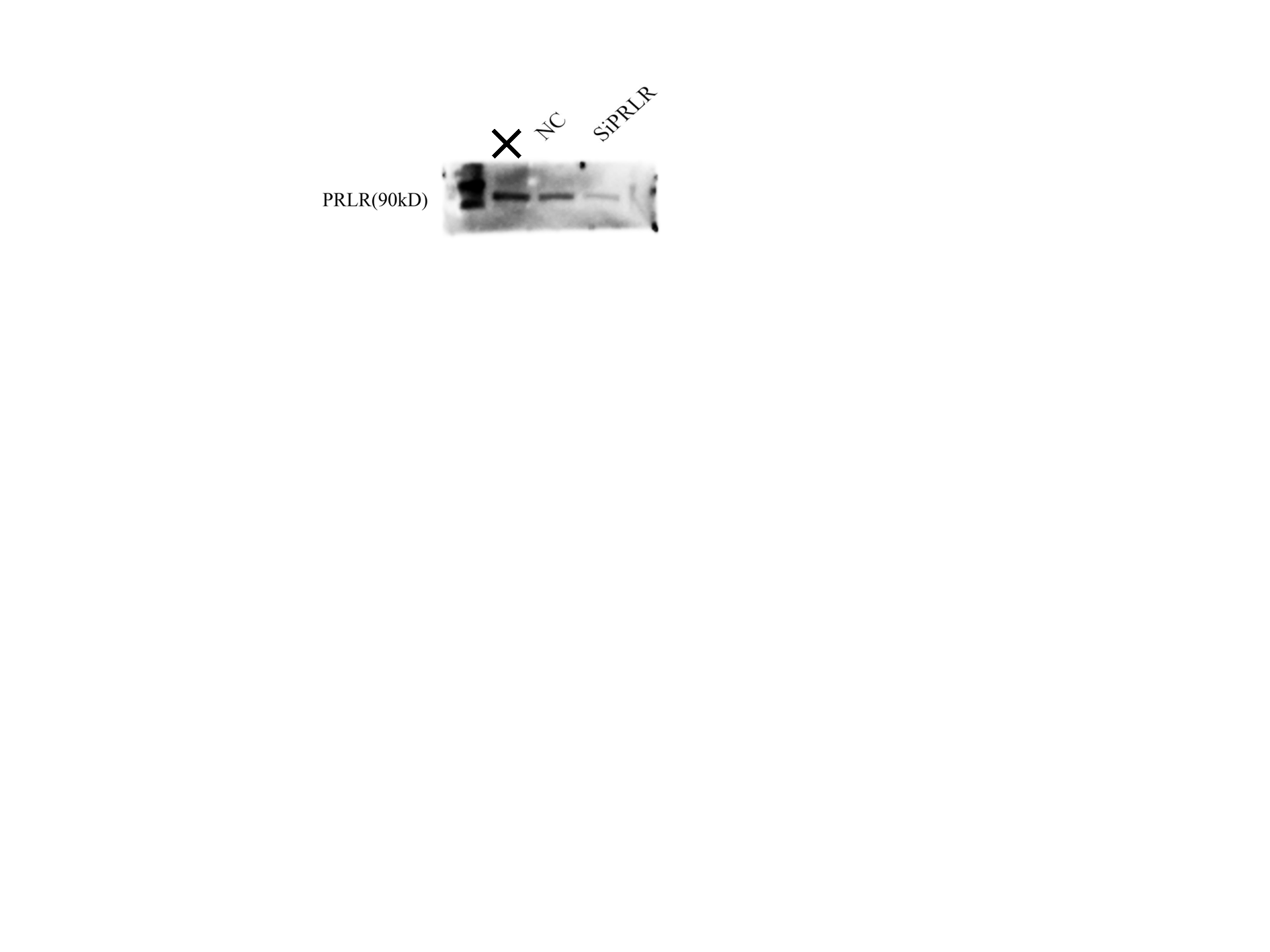

Supplement: Supplementary file 1 [file cells-13-01461-s001.zip › Original Images for Blots/Figure 8C/PRLR.tif]

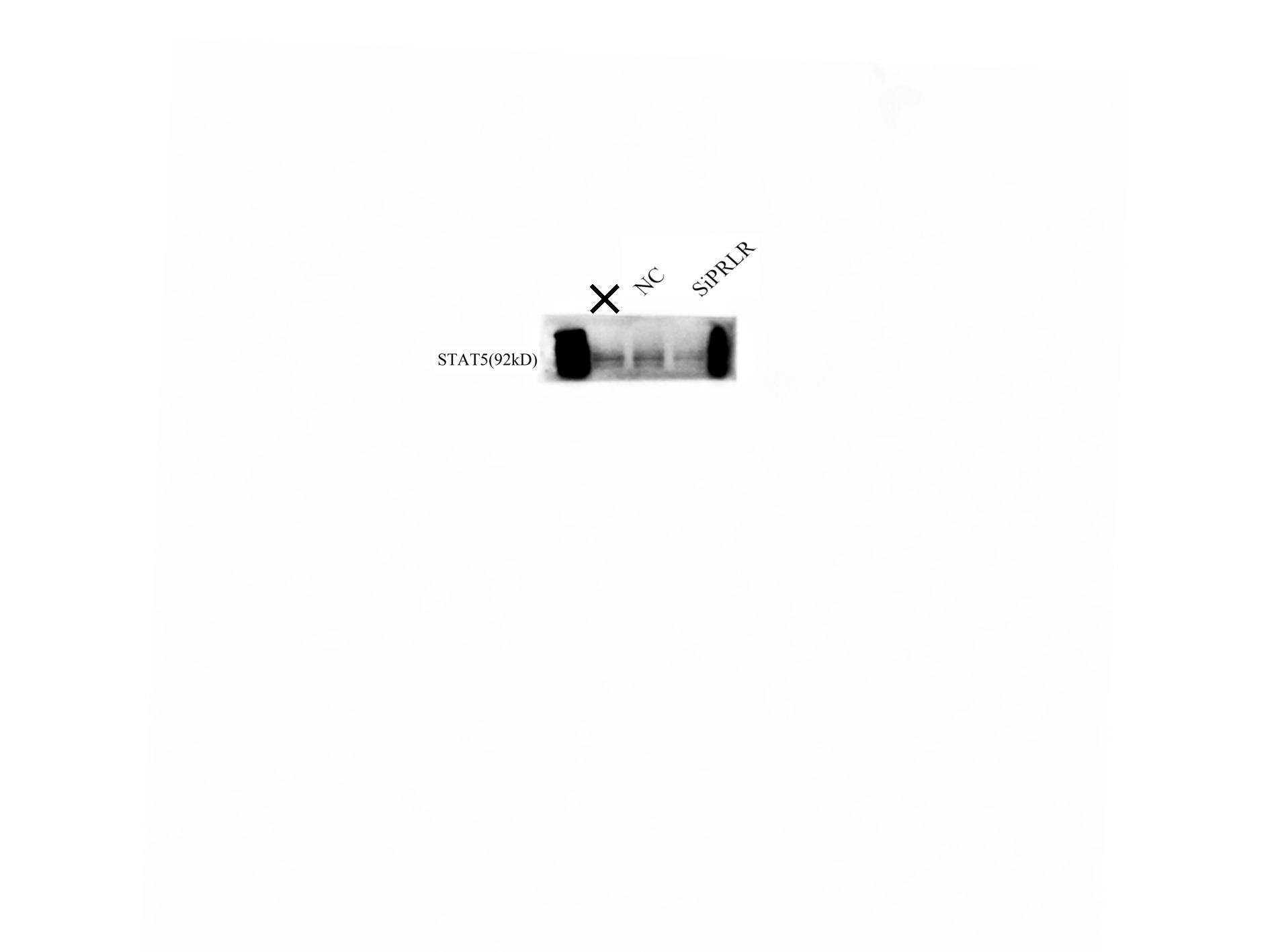

Supplement: Supplementary file 1 [file cells-13-01461-s001.zip › Original Images for Blots/Figure 8C/STAT5.tif]

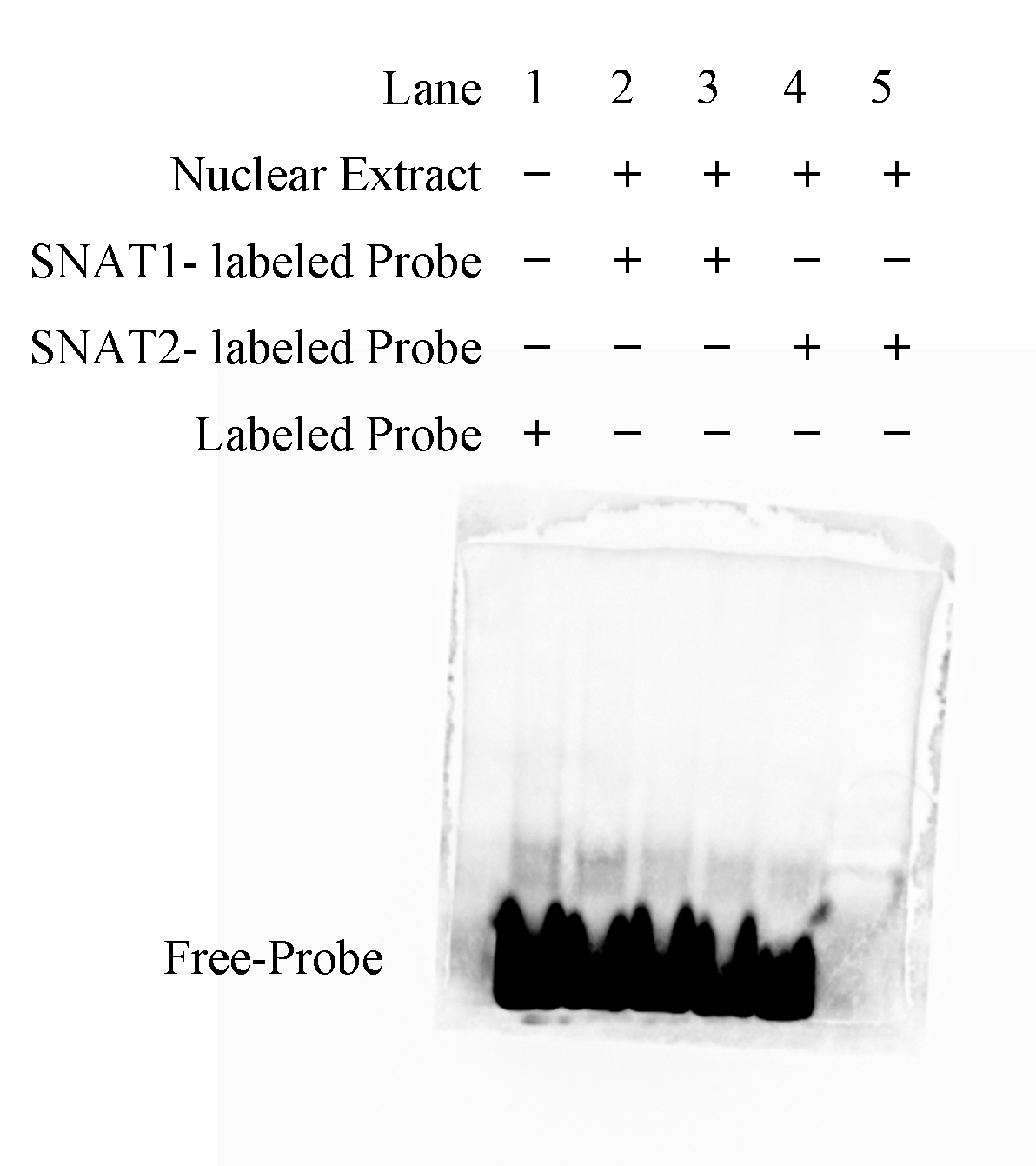

Supplement: Supplementary file 1 [file cells-13-01461-s001.zip › Original Images for Blots/Figure S3.tif]

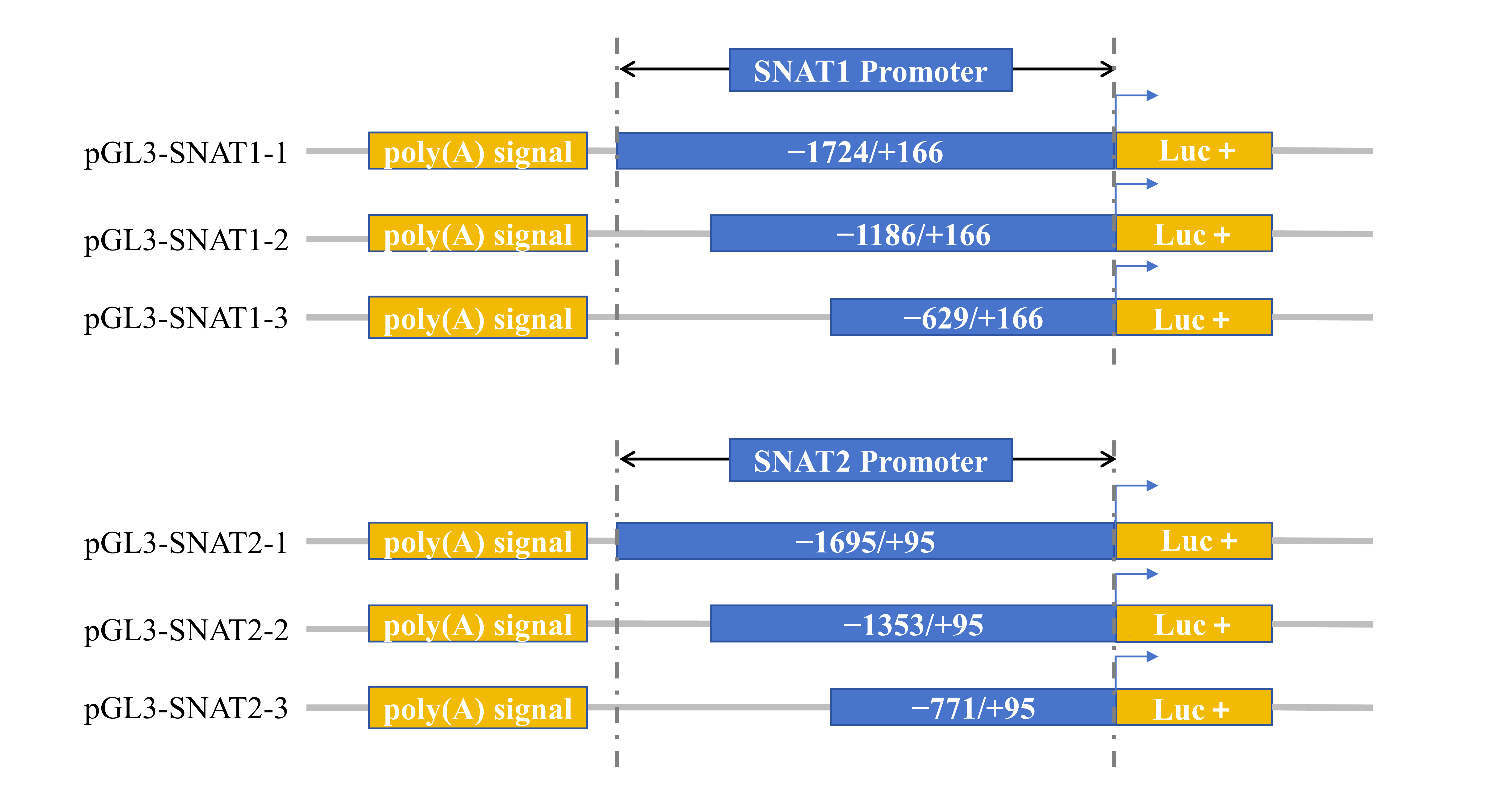

Supplement: Supplementary file 1 [file cells-13-01461-s001.zip › SUPPLEMENTAL INFORMATION/Figures-SUPPLEMENTAL INFORMATION/Figure S1.tif]

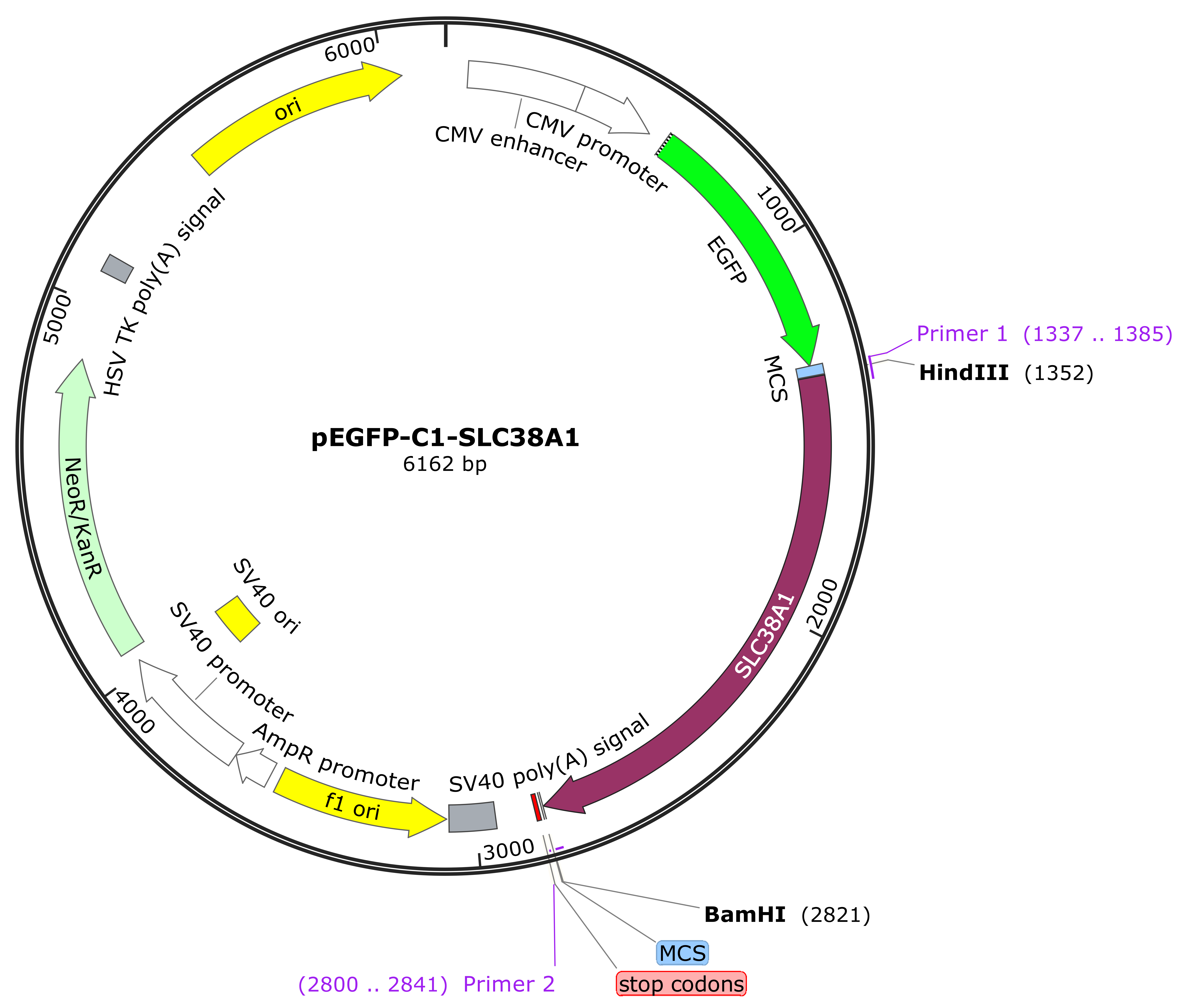

Supplement: Supplementary file 1 [file cells-13-01461-s001.zip › SUPPLEMENTAL INFORMATION/Figures-SUPPLEMENTAL INFORMATION/Figure S2.tiff]

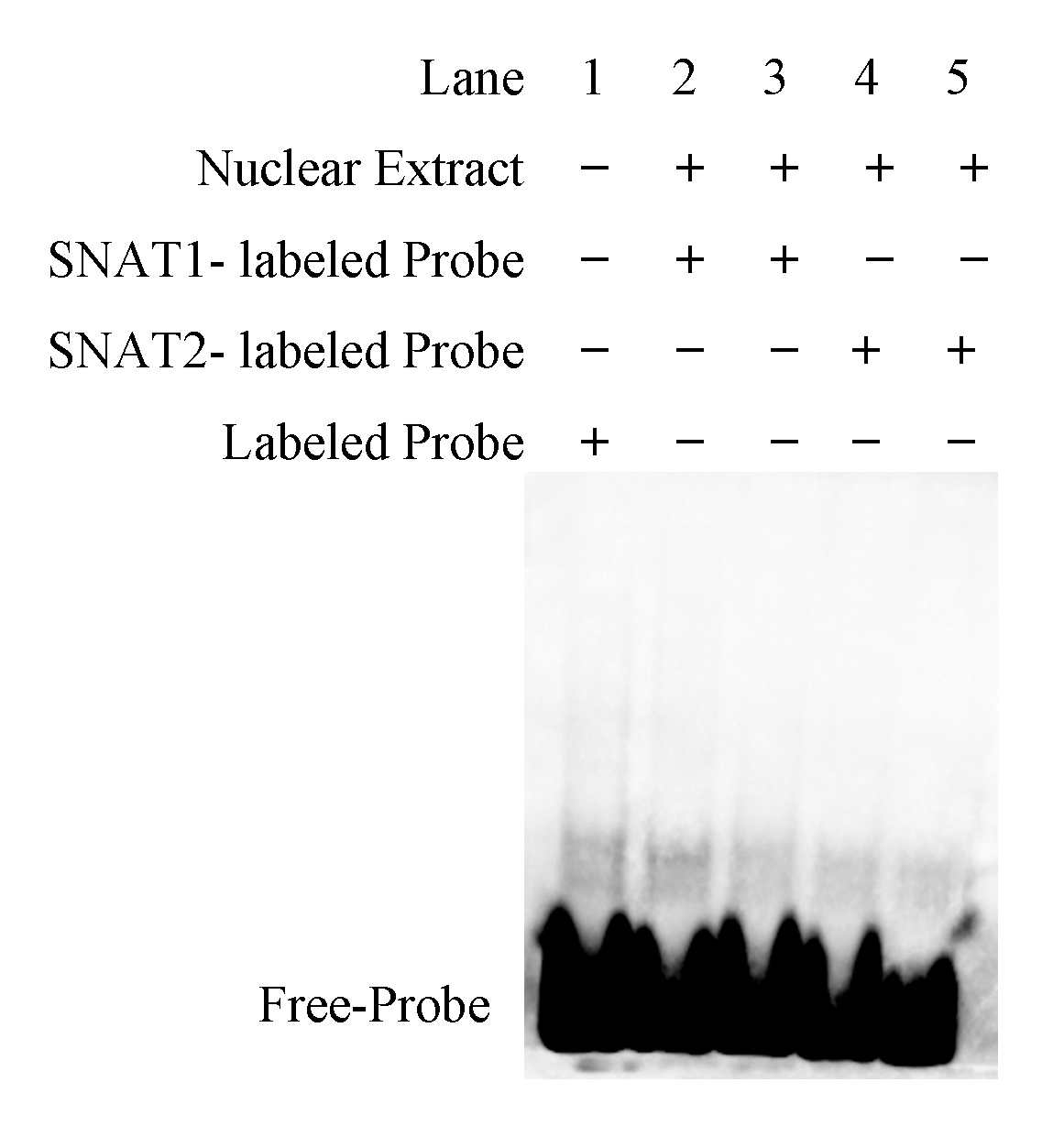

Supplement: Supplementary file 1 [file cells-13-01461-s001.zip › SUPPLEMENTAL INFORMATION/Figures-SUPPLEMENTAL INFORMATION/Figure S3.tif]
